# Directed Cortical Information Flow during Human Object Recognition: Analyzing Induced EEG Gamma-Band Responses in Brain's Source Space

**DOI:** 10.1371/journal.pone.0000684

**Published:** 2007-08-01

**Authors:** Gernot G. Supp, Alois Schlögl, Nelson Trujillo-Barreto, Matthias M. Müller, Thomas Gruber

**Affiliations:** 1 Department of Neurophysiology and Pathophysiology, Center of Experimental Medicine, University Medical Center Hamburg-Eppendorf, University of Hamburg, Hamburg, Germany; 2 Max Planck Institute for Human Cognitive and Brain Sciences, Leipzig, Germany; 3 Institute of Human-Computer Interfaces, University of Technology, Graz, Austria; 4 Intelligent Data Analysis Group, Fraunhofer Institute FIRST, Institute Computer Architecture and Software Technology, Berlin, Germany; 5 Cuban Neuroscience Center, Havana, Cuba; 6 Institute of Psychology I, University of Leipzig, Leipzig, Germany; Vrije University Amsterdam, Netherlands

## Abstract

The increase of induced gamma-band responses (iGBRs; oscillations >30 Hz) elicited by familiar (meaningful) objects is well established in electroencephalogram (EEG) research. This frequency-specific change at distinct locations is thought to indicate the dynamic formation of local neuronal assemblies during the activation of cortical object representations. As analytically power increase is just a property of a single location, phase-synchrony was introduced to investigate the formation of large-scale networks between spatially distant brain sites. However, classical phase-synchrony reveals symmetric, pair-wise correlations and is not suited to uncover the directionality of interactions. Here, we investigated the neural mechanism of visual object processing by means of directional coupling analysis going beyond recording sites, but rather assessing the directionality of oscillatory interactions between brain areas directly. This study is the first to identify the directionality of oscillatory brain interactions in source space during human object recognition and suggests that familiar, but not unfamiliar, objects engage widespread reciprocal information flow. Directionality of cortical information-flow was calculated based upon an established Granger-Causality coupling-measure (partial-directed coherence; PDC) using autoregressive modeling. To enable comparison with previous coupling studies lacking directional information, phase-locking analysis was applied, using wavelet-based signal decompositions. Both, autoregressive modeling and wavelet analysis, revealed an augmentation of iGBRs during the presentation of familiar objects relative to unfamiliar controls, which was localized to inferior-temporal, superior-parietal and frontal brain areas by means of distributed source reconstruction. The multivariate analysis of PDC evaluated each possible direction of brain interaction and revealed widespread reciprocal information-transfer during familiar object processing. In contrast, unfamiliar objects entailed a sparse number of only unidirectional connections converging to parietal areas. Considering the directionality of brain interactions, the current results might indicate that successful activation of object representations is realized through reciprocal (feed-forward and feed-backward) information-transfer of oscillatory connections between distant, functionally specific brain areas.

## Introduction

The involvement of gamma oscillations in the activation of cortical object representation is one essential finding of human electroencephalogram (EEG) and magnetoencephalogram (MEG) research. Regarding visual object recognition several studies reported a modulation of induced gamma-band responses (iGBR) by stimulus familiarity (e.g. [1]–[3]). Such iGBRs have been defined as electrical brain activity characterized by oscillatory bursts above 30 Hz and a jitter in latency from one trial to the next [4], [5]. The presentation of familiar objects leads to a stronger iGBR increase as compared to unfamiliar controls. This enhancement appears around 250 ms after stimulus-onset, depending on the time-point of object identification [6]. Based on reports from intracranial brain signals as well as from macroscopic scalp recordings, the varying level of gamma-power seems indicative of the formation of local neuronal assemblies implementing feature integration in the course of object identification [7]–[9].

In principle, a signal recorded by a single EEG-electrode represent the spatial summation of local-field-potentials (LFPs) of a large neuronal population, while local synchronization of their activities leads to frequency-specific power increase at this electrode [10], [11]. Thus, power changes alone cannot mirror the formation of large-scale networks that rest on oscillatory interactions between spatially distant cortical populations [12], [13]. This requires coupling measures such as phase-locking analysis (PLA), which was introduced on the basis of wavelet decompositions to measure long-range synchronization [14], [15]. By applying PLA to iGBRs, a high number of phase-lockings between scalp electrodes was revealed for familiar relative to unfamiliar objects [16]–[18]. Since phase-locking between scalp electrodes can be confounded by volume conduction artifacts, it is essential to know that intracranial EEG recordings from human cortex have demonstrated the physiological plausibility of phase-synchrony. In particular, unequivocal physiological evidence for the formation of large-scale interactions between distributed brain structures by means of long-range gamma synchrony has been obtained from intracranial recordings in humans (for a review see [19]).

In order to go beyond coupling analysis between scalp recording sites and to assess oscillatory interactions between brain areas directly, PLA was successfully applied in source space [20]. In brief, iGBR generators can be reconstructed by variable-resolution-electromagnetic-tomography, VARETA [21], [22]. Using this approach, iGBRs related to cortical object representation were localized to temporal, frontal and parietal brain areas [20], each reported to play a specific functional role in the cortical network mediating visual object recognition [23]–[25].

Here, we surpassed PLA by an advanced measure, partial-directed-coherence (PDC) based on multivariate-autoregressive modeling. In contrast to PLA, the multivariate PDC approach measures how several positions are ‘effectively’ connected (i.e. exclusively revealing direct connections by correcting for indirect influences), rather than merely describing pair-wise synchronicity. In particular, PDC captures the direction of information-flow by employing the concept of Granger-Causality in the frequency domain [26], [27]. The multivariate analysis of PDC evaluates each possible direction of brain interaction and reveals influences received from or transmitted by each brain area, and, consequently, even feedback influences can be uncovered. Since feedback seems to play a central role in neural communication, in particular in heavily interconnected brain structures such as the cortex, the potential benefit of applying directed coupling analysis becomes evident.

The goal of the present study is first to investigate, whether autoregressive modeling and wavelet analysis are equally suitable in detecting iGBRs elicited by visually presented objects. Secondly, we aimed to evaluate the connectivity pattern between the cortical brain sources underlying these induced gamma oscillations by calculating both coupling-measures, PLA and PDC. In particular, we sought to go beyond mere phase-locking changes by identifying the dynamic brain network of directed information-flow in activated cortical object representations.

## Results

**Figure 1:**
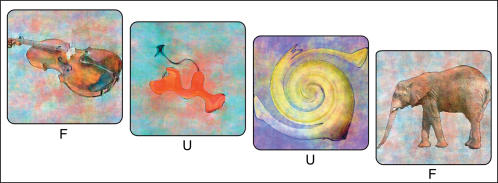
Excerpt of stimulus sequence. Familiar (F) and unfamiliar (U) color pictures were presented in randomized order.

Ten subjects were presented with pictures of familiar and unfamiliar objects (see Figure 1 for some sample pictures) and asked to categorize them, while EEG signals were recorded from 128 channels and stored for offline analysis. Behavioral data revealed about 97 percent of correct answers, i.e. participants correctly categorizing a visually presented pictorial image either as familiar (meaningful) or unfamiliar (meaningless). The low percentage of errors underlines the usability of the current paradigm in eliciting brain processes related to object recognition.

### Autoregressive modeling: spectral power changes – electrode space

**Figure 2:**
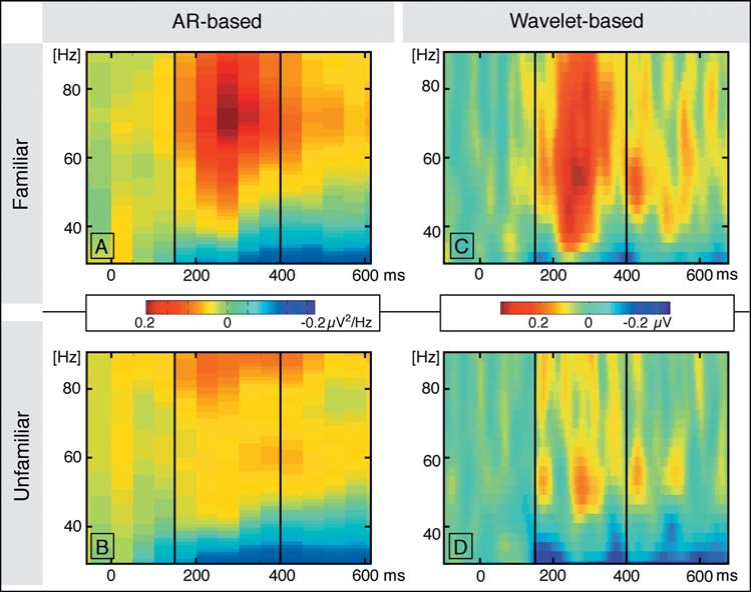
Induced spectral changes within the gamma-band range represented by time-frequency (TF) plots for each condition (familiar and unfamiliar). TF plots A and B are based on autoregressive modeling (AR), C and D on Morlet wavelet analysis. The two vertical black lines indicate the time interval of the induced gamma-band peak (150–400 ms post-stimulus onset) as used for further analyses. All TF plots were baseline corrected, averaged across subjects and twenty-two parieto-occipital electrodes (*cf*. Figure 3).

**Figure 3:**
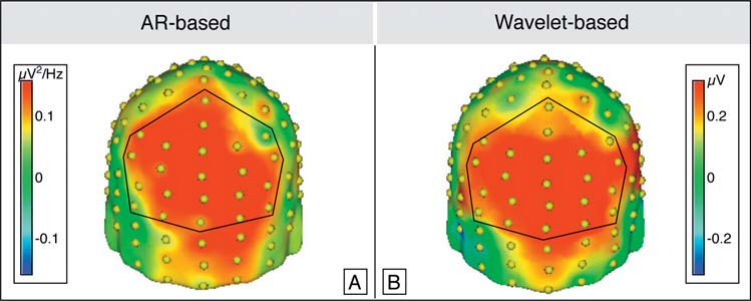
Grand mean spherical-spline interpolated topographies of the condition effect (familiar minus unfamiliar) as revealed by AR (autoregressive) modeling (A) and wavelet analyses (B). Both maps are based on the induced gamma-band peak from 150 to 400 ms after stimulus onset. Electrodes as used for TF plots and statistical analyses are hemmed by black lines.

The spectral changes based on the applied autoregressive model (AR) within the iGBR range are represented in Figure 2A and 2B. The baseline-corrected time-frequency (TF) plots averaged across 10 subjects and 22 electrodes (clustered to form a parieto-occipital region of interest: see Figure 3) are depicted separately for each condition. Baseline-corrected spectral power induced by familiar object presentations showed a clear peak in a time window from 150 to 400 ms after stimulus onset in a frequency range between 40 and 90 Hz. Although the increase was present even beyond 90 Hz, we restricted our analysis to the range mentioned above in order to compare the results with the wavelet approach. Statistical analysis revealed a higher iGBR increase for familiar as opposed to unfamiliar objects (t(9) = 12.4; p<0.0001). A difference topography map of this effect (familiar minus unfamiliar) is depicted in Figure 3A. A broad posterior distribution with a maximum at parietal and occipital electrode sites can be appreciated.

### Wavelet analysis: spectral power changes – electrode space

Figure 2C and 2D depict the wavelet-based baseline-corrected TF-plots for each experimental condition averaged across all subjects and all electrodes of a central-posterior regional mean. IGBR increases elicited by familiar objects revealed a clear peak in a time window from 150 to 400 ms after stimulus onset in a frequency range between 40 and 90 Hz (Figure 2C). This increase is significantly higher for familiar as opposed to unfamiliar objects (t(9) = 6.2; p<0.001). A topographical difference distribution of the iGBR peak (familiar minus unfamiliar) is depicted in Figure 3B. The effect shows a broad posterior scalp distribution with a maximum at parietal and occipital electrode sites. Importantly, convergent topographies of the familiarity effect are obtained through each analysis technique, wavelet decomposition and autoregressive modeling. Given the maximum at parieto-occipital electrodes and the lack of a frontal effect in iGBRs we displayed the difference topography maps from a posterior point of view.

Note that the wavelet-based TF representations show a more refined time course of the iGBR as opposed to AR spectral results, because AR modeling presupposes a sufficiently long data window for analysis (see Materials and Methods, Section A for details). Furthermore, the iGBR peak in the AR-based TF-plot (72 Hz) reveals a displacement relative to the one found by wavelet analyses (around 58 Hz). However, this does not indicate differential peak frequencies, because the power spectral density derived from the AR parameters is characterized by a center frequency (i.e., 72 Hz) and its edge frequencies (+/−21 Hz). The peak as obtained by wavelet analysis lies within this range given by the center frequency and these limits. Therefore, both measures have revealed comparable findings.

### Information transfer: partial-directed coherence (PDC) in source space

**Figure 4:**
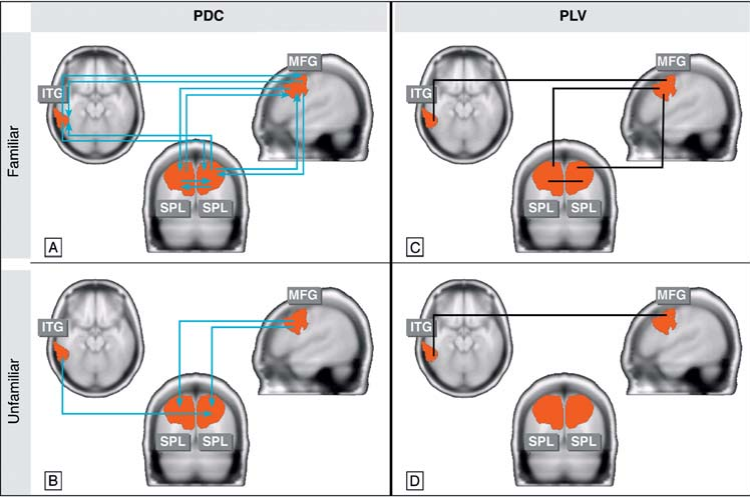
Tomographies and coupling patterns of the induced gamma-band peak elicited by familiar and unfamiliar stimuli (150–400 ms after stimulus onset). In the SPMs significant differences (familiar versus unfamiliar) are indicated in red. The following Regions of Interest (ROIs; *cf*. Table 1) were defined: ITG (inferior-temporal gyrus, left), SPL (superior-parietal lobe, bilateral), MFG (middle frontal gyrus, right). The arrows in A and B represent the direction of information transfer between the ROIs and were only drawn if the PDC values were significant (p<0.001). The lines in C and D display significant increases of phase-locking values (p<0.001) calculated between all ROIs.

Figure 4A and 4B depict the results of PDC analysis between four cortical areas (Regions of Interest; ROIs) for familiar (A) and unfamiliar (B) objects in a time window from 150 to 400 ms after stimulus onset. The ROIs were defined based on the statistical-parametric-maps (SPMs) of the condition effect of the iGBR peak (see Materials and Methods). The centers of gravity for these four brain locations are listed in Table 1 and are depicted as spots of significant activations at the respective anatomical location. The pattern of significant PDC values calculated between the investigated brain sites revealed quantitative and anatomical differences in information transfer during the presentation of familiar and unfamiliar objects. Significant PDC values between iGBR generators are indicated by arrows that represent the direction of information transfer (p<0.001).

**Table 1:** MNI (Montreal Neurological Institute) coordinates and anatomical descriptions of the centers of gravity of all Regions of Interest (ROIs) associated with the condition effect (familiar *versus* unfamiliar) on the induced gamma-band response (150–400 ms after stimulus onset).

Region of Interest	Anatomical description	MNI coordinates of the center of gravity		
		x	y	Z
ROI 1	inferior temporal gyrus – left (ITG)	−57	−48	−17
ROI 2	superior parietal lobe – left (SPL)	−21	−69	34
ROI 3	superior parietal lobe – right (SPL)	21	−69	34
ROI 4	middle frontal gyrus – right (MFG)	50	17	35

Note that the inverse solutions of the effect at both parietal areas are distinctly separated from another. The maxima are located in the very center of each ROI (left-SPL and right-SPL, respectively). Thus, the possibility of a single centro-parietal source is excluded.

**Figure 5:**
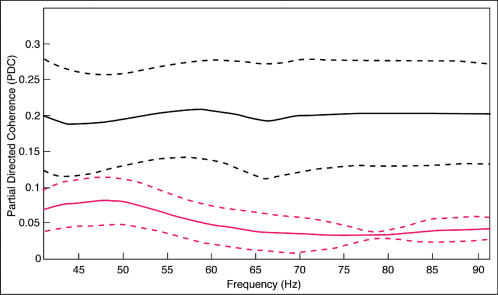
Mean partial directed coherence (PDC) values computed over all significant ROI pairs for each condition (solid black line: familiar; solid magenta line: unfamiliar). The dashed lines represent the corresponding standard errors of PDC values.

The number of significant PDC values during familiar object presentations surpasses that for the meaningless condition (ten versus three). Furthermore, during processing of familiar objects the network of information transfer is bidirectional. Each area transmitting information towards a given brain site receives information input from it as well. In contrast, unfamiliar objects elicit a sparser number of significant PDC couplings, all which are unidirectional and converge towards parietal brain areas (Figure 4B). The actual course of PDC values from all significant brain interactions is represented in Figure 5 for each condition separately.

In order to investigate the consistency of the reported couplings and to clarify to what extent the reported connections depend on a certain statistical threshold applied, we have repeated the statistical analysis with several different thresholds.

The results summarized in Table 2 indicate that identical coupling patterns (in terms of number and coupling pairs involved) were obtained between p<0.001 and p<0.02, suggesting stability over a considerable range of statistical thresholds. In fact, those coupling patterns that appeared at our originally chosen threshold of significance (i.e. p<0.001) remained unchanged up to a p-level of 0.02. Only with a threshold of p<0.05 additional connections become significant in both conditions. However, in order to take into account the considerable number of comparisons tested (with 4 positions giving 4×3 = 12 possible combinations) and to avoid spurious positives, it seems necessary to apply a lowered threshold (in our case: p<0.001) for statistical analysis of coupling results (for a similar approach see [28]–[30]). According to the Bonferroni correction method the fact of multiple comparisons needs to be corrected by a lowered p-value, i.e. p<0.004 (0.05/12), a threshold which revealed identical coupling patterns as those displayed in Figure 4. Therefore, the pattern of connections represented here stood up to rigorous statistical correction.

**Table 2:** Number of significant PDC and PLA couplings using different statistical thresholds.

Statistical Threshold	Number of significant couplings			
	PDC (familiar objects)	PDC (unfamiliar objects)	PLA (familiar objects)	PLA (unfamiliar objects)
p<0.001	10	3	4	1
p<0.01	10	3	4	1
p<0.02	10	3	4	1
p<0.05	11	8	4	3

The number of connections found to be significant at a given level of statistical threshold applied is listed separately for each coupling measure and each experimental condition.

### Long-range synchronization: phase-locking analysis (PLA) in source space

Figure 4C and 4D depict the results of phase-locking analysis (PLA) between all four ROIs (see Table 1) for familiar and unfamiliar objects in a time window from 150 to 400 ms after stimulus onset. For familiar object presentations significant phase-locking (i.e. p<0.001) was established between most of all possible ROI-combinations (i.e. four out of six possible couplings). In contrast, unfamiliar objects were associated with far less significant phase-locking values, leaving just one pair significant. The coupling pattern of PLA proved to be consistent over a range of different thresholds of significance, as demonstrated by the results listed in Table 2.

For both experimental conditions we have evaluated the phase angles at which synchronization occurred and we found that in the familiar case they were centered on a mean of 0.2 rads (std error: 0.1 rads). In the unfamiliar case, the distribution was broader (mean = 0.01; std error 0.2 rads). Since artifactual influence of volume conduction causes only phase-locking with zero phase-lag, our patterns of phase-locking for familiar object presentation cannot solely be explained by volume conduction.

## Discussion

Natural objects are composed of numerous lower and higher-level features, which are cortically represented in dispersed brain areas. Synchronized oscillatory neuronal activity in the gamma frequency range (>30 Hz) is regarded as a plausible mechanism to integrate these features into a coherent percept (for reviews, see [2], [3], [31]). Previous studies have suggested that integration of stimulus features activates a respective memory content (for a detailed discussion see e.g. [9], [32]). The functional link between sensory processes and memory has been established by empirical work (for some reviews see: [33]–[35]) and a detailed account on how sensory feature processing my give rise to the emergence of high-level representation (semantic features) has been proposed recently [36].

Whereas previous publications successfully demonstrated synchronicity between source-reconstructed generators of induced gamma-band responses, iGBRs [32], the present study was designed to go one crucial step further. In particular, we intended to unravel the *causal* connectivity between the brain sources of iGBRs by using the measure of partial-directed coherence (PDC), which is based on multivariate-autoregressive modeling. Additionally, we intended to contrast these findings with ‘conventional’ wavelet-based phase-locking results and to validate the functional plausibility of our results by relating the network of directional brain interactions with experimental findings from other methodologies.

To induce robust gamma-band oscillations, we applied a standard object recognition paradigm in which familiar and unfamiliar visual stimuli are presented [1], [16], [20]. At the scalp level, both techniques (autoregressive modeling and wavelet analysis) replicated previously reported increases of iGBRs during the presentation of familiar relative to unfamiliar objects [5], [16], [32]. Both approaches revealed comparable results in terms of latency (150–400 ms), topography and frequency of the iGBR peak (wavelet-based: 58 Hz and AR-based: 72 Hz; see Methods for details). The time range of the iGBRs corresponds well with previous studies making use of an object recognition paradigm, reporting 150 to 400 ms and 200–400 ms (post-stimulus onset), respectively [1], [20]. On the whole, AR modeling is well suited to detect induced and transient high-frequency oscillations in the human EEG.

Concerning the presented tomographical analyses, our procedure has been suggested by and is in agreement with several EEG/MEG source localization studies that have investigated the neuronal generators underlying frequency-specific power changes (e.g. [20], [37]–[39]). In particular, our results are in line with previously reported iGBR generators [20], [40] localized in four anatomically dispersed cortical areas, between which a dense pattern of synchronicity was established in response to familiar objects analyzed by means of conventional phase-locking analysis (PLA; [14]). In contrast, hardly any significant phase synchronization was established during the presentation of unfamiliar objects.

The pattern of causal connectivity (PDC) related to the processing of familiar objects resembles the coupling results based on phase-synchrony (PLA) in terms of its overall connectivity. Equally important, the fewer number of significant couplings for unfamiliar objects (in relation to familiar ones) are reflected by both measures. Thus, in principle, both techniques revealed a highly convergent pattern of brain connectivity during object recognition. As the dependence of coupling results on the applied statistical threshold is concerned, the numbers of significant connections are listed in Table 1 for a range of several thresholds. The pattern of connectivity displayed in Figure 4 for both measures, PDC and PLA, have proofed to stay identical in the face of a considerable range of statistical thresholds applied (between p<0.001 to p<0.02).

By comparing PDC and PLA results in a qualitative sense, the PDC connectivity pattern for unfamiliar stimuli shows to differ from the one obtained by PLA. This fact underlines that PDC does not merely reflect instances of phase-synchrony but rather represents a methodologically distinct measure that quantifies the temporal dependencies between brain signals and, therefore, assesses influences received from or transmitted by each brain area. That is, differences between coupling patterns obtained by PDC and PLA are rooted in the fact that different aspects of the underlying signals are reflected in each measure. While PLA is a symmetric measure of (phase) relations inside a pair of signals, the multivariate measure of PDC was developed to reveal the temporal precedence, i.e. the causal hierarchy between activities. As an important consequence, whenever feedback between signals exists, simple correlation measures may not capture such dependencies, while PDC was introduced to overcome this limitation and specifically should reflect temporal feedback relations [26], [41]. Furthermore, PLA is calculated for each pair separately (bivariate analysis) and does not differentiate between direct and indirect (phase) couplings, so that both types of relations influence the actual PLA value. In contrast, the multivariate approach of PDC is suited to characterize solely direct dependencies of two signals under study. Expressed in qualitative terms, this is possible since any signal providing a common influence to the interaction under scrutiny (originating from other signals within the multivariate time-series) are not taken into account, i.e. common sources are partitioned or separated, so that they do not enter the determination of PDC values [42], [43]. Therefore, PDC values should reflect direct interactions in particular, a fact that is also referred to as ‘effective connectivity’.

The directionality of these interactions as extracted by PDC and their possible significance will be addressed after we have highlighted the possible functional role of the brain areas identified. In this context we will also discuss empirical evidence from other studies supporting the functional plausibility of the limited PDC pattern elicited by unfamiliar objects.

The iGBR generators associated with object representation were identified at left inferior temporal, right prefrontal and bilaterally at superior parietal brain areas. Supporting evidence from lesion studies, intracortical recordings and functional neuroanatomy suggest the involvement of these areas in visual object processing. Neuronal populations within the inferior temporal cortex, a brain structure known to be part of the ventral visual stream, has been found to be tuned to relatively complex relations among elementary visual features [25], [44], [45]. Frontal activation has been reported in several studies to represent top-down facilitation during object recognition [23], [46]–[48]. Superior parietal cortex has been repeatedly linked to feature binding, in the sense that lower-level object features have to be spatially integrated to form a visual object [24], [49], [50]. These reports are in line with the general idea proposing that object recognition is a cooperative process resulting from a interlinked set of brain areas (for a review, see [51]). Such cooperative processes forming functional networks are particularly suited to be investigated by coupling measures such as phase-locking analysis (PLA) or partial-directed coherence (PDC). The present PDC pattern in response to familiar pictures might reflect a more intense network of interactions between cortical regions that is initiated by the integration of functionally specialized areas associated with object representation [15], [18], [20]. Importantly, due to the directional property of the PDC measure, this network of information transfer is found to be exclusively realized by bidirectional connections. In fact, this result is to be expected on theoretical and functional grounds, since the temporal coordination of input-triggered responses and their integration into functionally coherent assemblies are presumably based on dynamic, distributed grouping through iterative reentry [13], [52]–[54].

Conversely, the small number of significant information flow during meaningless object processing was all one-sided, possibly indicating unidirectional communication in the sense that one brain side constitutes the oscillatory drive of the other [13], [52], [55]. The restricted number of brain interactions might be due to the fact that no representation can be activated in areas relevant for structural integration of object features. Since unfamiliar objects contain lower-level features similar to the ones in our familiar objects (such as low spatial frequencies), but lack meaningful structural information, the sparse connections of information transfer converging at parietal areas might reflect the processing of these isolated object features. Specifically, the frontally originating information transfer might be due to top-down influence that is assumed to be initiated by low-spatial frequencies also contained in unfamiliar stimuli [23], [47]. The activation of the inferior temporal cortex, providing input towards parietal cortex, could be expected in the face of the reported preferential responses of this brain structure to relatively complex relations among elementary visual features equally provided by unfamiliar objects [25], [44]. The functional plausibility of our directional coupling results during meaningless presentation (with parietal areas being the converging site) is further supported by a recent study on face perception [56]. The authors demonstrated a parietal increase of iGBRs in response to correctly configured components of a human face as compared to stimuli in which the different features of a face were presented at atypical locations lacking a coherent representation and failing to induce a respective integration process.

We have to point out that there is still another, alternative interpretation that may account for the sparse number of significant connections during unfamiliar object processing. Instead of concluding a reduced level of brain interactions from a small number of couplings, it is equally possible that a local reduction in gamma activity (i.e. a reduction of short-range synchronization) gives rise to sparse number of couplings without changing the underlying interactions between brain areas. That is, a reduction in PDC and PLA couplings in the unfamiliar condition might reflect merely a reduction in localized gamma activity. Essentially, this is due to the fact that all types of coupling measures (including PDC and PLA) are invariably sensitive to changes of signal-to-noise ratio (SNR). For this reason, at present we can not rule out this alternative interpretation for the coupling pattern of the unfamiliar condition, neither theoretically nor experimentally. To discard this alternative interpretation further methodological developments have to address this issue in depth. Another, possibly straight forward solution is to compare two experimental conditions, each eliciting a similar level of frequency specific local neural responses, by contrasting the directionality of communication between both conditions directly.

Our current work was aimed to go beyond mere phase-locking changes by using the advantage of the Granger-causality-based multivariate-autoregressive models (MVAR) of PDC providing a frequency-specific measure of directional interactions [26], [57]. Several other methods have been proposed to obtain electrophysiological patterns of brain connectivity on the basis of estimated cortical activity (for reviews, see [39], [58]). Noteworthy, another MVAR coupling measure has been developed, namely directed-transfer function (DTF), which is analytically highly related with PDC [27], [59]. As PDC, also DTF complies with the necessity of using a multivariate approach as opposed to pair-wise calculation in assessing the information flow between physiological time series [59], [60]. Over the recent years, PDC and DTF have received growing attention in electrophysiological research and have been studied under several simulation conditions (e.g. [57], [59], [61]), and also have been investigated in source space (e.g. [62]–[64]). However, the localization of sources in these studies was restricted to those Brodmann areas that were pre-selected on anatomical grounds together with a-priori assumptions regarding the functional role a given cortical brain area might play. In contrast, our source reconstruction was solely guided by localizing the oscillatory effect in the gamma frequency band, i.e. the induced gamma power changes modulated by the familiarity of the stimuli. This kind of source localization was crucial for our investigation, since we sought to identify the brain areas giving rise to iGBRs, i.e. that underlie the process of visual object representation, in order to characterize the functional network established among those areas.

A challenging future perspective is certainly to investigate more complex cognitive processes such as e.g. working memory or constituting parts of explicit and implicit memory networks and the distinct directionality of their interactions. These findings could be strengthened by data from intracranial recordings (e.g. [65]–[67]).

### Conclusion

The present study is the first to identify the directionality of oscillatory brain interactions in source space during human object recognition and demonstrate that familiar, but not unfamiliar, objects engage widespread reciprocal information flow. The multivariate PDC coupling approach brings a qualitative improvement over traditional phase-locking analysis by delivering the directionality of brain interactions. The distinct reciprocity of the PDC coupling pattern in response to familiar visual objects provide experimental evidence for the idea that functional brain networks, successfully implementing object feature integration, are realized by extensively reciprocal (feed-forward and feed-backward) oscillatory interactions between specific brain areas [13], [52]–[54]. Unfamiliar stimuli might fail to elicit such an integration mechanism, so that the solely unidirectional PDC couplings possibly could reflect the restricted processing of isolated object features.

## Materials and Methods

### Participants

Ten healthy, right-handed university students (7 female; aged 20 to 27 years, mean: 23.6, SD: 2.2) were paid for participation (6 EURO per hour). The experimental protocol conformed to local ethics guidelines (ethics board of the University of Leipzig) and the Declaration of Helsinki. Participants gave informed consent prior to the start of the experiment. All participants had normal or corrected to normal vision and had no recorded history of neurological or psychiatric disorders.

### Stimuli and procedure

Experimental stimuli were either color pictures, selected from a standard picture library (Hemera Technologies, 1997), representing real-life objects such as apple, cup or elephant (i.e. *familiar* or meaningful objects, n = 200) or color pictures of *unfamiliar* objects (i.e. meaningless objects, n = 200) – see Figure 1 for some examples. The pictures of unrecognizable objects were created by the authors through distorting meaningful images from the library such that they physically matched the meaningful pictures in every possible way (e.g. size, complexity, part-structure) except for familiarity. A detailed description of the distortion procedure can be found elsewhere [1].

Two experimental lists were created from the stimulus pool for each subject by randomly choosing 100 familiar and 100 unfamiliar pictures. A different picture was presented in every trial in order to avoid previously reported repetition suppression effects of the iGBRs [68], [69]. All stimuli (approx. 6 by 6 degrees) appeared in randomized order and were presented centrally on a 19″ CRT-screen (refresh rate: 70 Hz) placed 1.5 meters in front of the subjects.

The temporal sequence of events within each of the 200 experimental trials was as follows: Preceding every item, a fixation cross (0.3 by 0.3 degrees) appeared on the screen for a randomized interval between 500 to 700 ms. The following presentation of each picture lasted for 700 ms and was replaced by the appearance of a fixation cross lasting for 800 ms. Responses had to be delivered at the end of a trial, which was indicated by the presentation of a query. In brief, the order of events within one trial was as follows: fixation – picture – fixation – query. The subject was required to respond whether the presented picture was a familiar or an unfamiliar entity by pressing a button with the respective index finger. The response-button-to-task allocation was counterbalanced across subjects. The subjects were asked to avoid movements and eye blinking during the presentation of the fixation cross and the visual objects. To allow for a short break the experiment was divided into two blocks of 100 trials each.

### Electrophysiological recordings

The experimental data have been gathered in an independent EEG study. However, it needs to be stated that this study used the same paradigm and the identical stimulus-set as reported previously [1]. The EEG was recorded from 128 Ag-AgCl electrodes positioned on the scalp with a BioSemi Active-Two amplifier system in an electrically shielded and sound attenuated room. Additionally, horizontal and vertical electrooculogram (EOG) were recorded to facilitate subsequent artifact detection resulting from eye-movements and blinks. EEG and EOG were sampled at 512 Hz. The EEG signal was high pass filtered (5^th^ order sinc response with a −3 dB point at 128 Hz) and stored for offline analysis. Two additional electrodes near channel CPz (CMS-Common Mode Sense and DRL-Driven Right Leg; cf. http://www.biosemi.com/faq/cms&drl.htm) were used as reference and ground, respectively. For further analysis the average reference was used. An automatized artifact correction was applied on EEG epochs starting 500 ms prior and 1500 ms following picture onset by means of “statistical correction of artifacts in dense array studies” (SCADS; [70]). This procedure is widely accepted in the field and was applied and described in several publications (e.g. [71], [72]).

### Data analysis (A): spectral power changes analyzed by autoregressive modeling

Changes in iGBRs were analyzed by means of autoregressive modeling. All following computational steps (for autoregressive modeling and PDC analysis) are implemented in BioSig (version: 1.95), an open source software library for biomedical signal processing, which is available on-line under http://biosig.sf.net [73].

Autoregressive (AR) modeling is an approach to time-series analysis by which a mathematical model is fitted to a sampled signal. AR modeling implies the value of the current sample *y(t)* in a data sequence of length N, *y(1), y(2), …, y(N)*, to be predicted by a linearly weighted sum of the *p* most recent sample values, *y(t−1), y(t−2), …, y(t−p)*, with *p* being the model order. If *y(t)* denotes the predicted value at time point *t* for a single channel, the AR model for this univariate case is formalized as
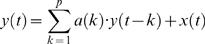
(1), whereby each past sample value *y(t−k)* is multiplied by the *k*-th autoregressive parameter *a(k)*, also termed regression coefficient. To complete the AR model a zero-mean white noise process, the “innovation process” *x(t)*, is added to the linear function [57]. The term *x(t)*, also often addressed as “prediction error”, is equal to the difference between the prediction derived from the linear combination of the most recent proceeding values (samples) and the actual value at time point *t*. In fact, this innovation process can not be equated with an error term in its usual sense, since according to the definition the modeled time series y*(t)* would be zero if *x(t)* is zero. Accordingly, *x(t) has* to be considered as the driving force of the model [74]–[76]. To estimate the AR parameters we used the Burg algorithm that was shown to be advantageous over other estimators [77]. In agreement with previous studies the model order *p* that defines the AR spectral resolution was set to 15 in order to guarantee a suitable resolution of several frequency components (i.e. *p*/2 = 15/2) in the subsequent analysis [74], [78], [79]. We opted for this way, after we had tried to find the optimal model order by the use of the Akaike Information Criterion, AIC [80] or of the Schwarz's Bayesian Criterion, SBC [81]. Our attempt to determine the optimal model order by locating the minimum of the AIC and SBC as a function of model order (*p* investigated between 2–30) revealed no consistent solution. In fact, AIC and SBC dropped monotonically with increasing model order, lacking any local minimum in the investigated interval. Therefore, in correspondence to previous EEG studies (see above), we selected a model order of 15, which can be regarded as a tradeoff between sufficient spectral resolution and overparameterization (for a similar approach see [82]). To obtain the spectral characteristics of the underlying signal the AR model is transformed into the frequency domain, where the power-spectral density (PSD) function for a given channel is derived as follows:
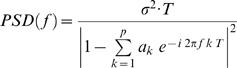
(2)Here, the variance of the innovation process is represented by *σ* ^2^ and *T* denotes the sampling interval (T = 1/*f_0_*; *f_0_* = 512 Hz). Importantly, for the present purposes, each trial was subdivided into 150 ms time windows overlapping by 50 ms, starting from −200 to 650 ms relative to stimulus onset, resulting in 77 samples *per* window. Subsequently, the information within each time window in each trial was concatenated resulting in one data stream to which one AR model was fitted. In other words, one AR model was fitted to a window of sample length equal to 77 times *N* trials that was consecutively moved in time by 25 samples (i.e., 50 ms). Note that trials within the created data stream were separated by a sufficient amount of not-a-numbers (NaNs), i.e. p+1 (15+1) number of NaNs preventing spurious correlations between trials. In total, 18 overlapping time windows of 150 ms length each were obtained, starting from 200 ms before up to 650 ms after stimulus onset. This approach reveals a finer resolved time course as opposed to the modeling of non-overlapping windows. Furthermore, due to the short analysis window the quasi-stationarity of the time-series is approximated [27], [83].

In order to identify the latency and frequency range of the iGBR peaks, the AR derived spectral power of the baseline (−200 to −50 ms prior to stimulus onset) was subtracted from the power values of all following time windows. Subsequently, these baseline-corrected power values, averaged across twenty-two parieto-occipital electrode sites and all subjects, were represented in separate time-frequency (TF) plots for each condition in the 30–90 Hz frequency range. The electrodes used for the TF-plot were selected on the basis of a spherical spline interpolated topographical distribution [84] of the gamma peak averaged across both conditions (for a similar approach see [1], [20]). The respective electrode sites are indicated in Figure 3A and 3B. For further analysis, the spectral power in the interval of maximum-induced gamma amplitudes (150–400 ms after stimulus onset, see Results) extending over three adjacent time windows was averaged for each subject. The resulting data from the parieto-occipital regional mean were analyzed by a paired t-test to determine whether gamma power differed significantly (p<0.001) between familiar and unfamiliar object presentations.

### Data analysis (B): spectral power changes analyzed by wavelet analysis

In order to compare the technique described in (A) to ‘conventional’ methods of frequency analyses we used a Morlet wavelet decompositions with a width of 7 cycles *per* wavelet. This approach has been exploited in a great number of EEG studies, since its introduction by Bertrand and Pantev in 1994 [85] (e.g. [14], [18], [32], [86]). For wavelet analysis (and subsequent source analysis) in-house procedures running under MATLAB (The MathWorks, Inc.) were used. Wavelet analyses result in a time-varying magnitude of the signal in each frequency band, leading to a time by frequency (TF) representation of the input. TF amplitudes are averaged across single trials, allowing one to analyze non phase-locked components. To exclude phase-locked values from the analysis, the evoked response (i.e. the ERP) was subtracted from each trial, similar to previous publications (e.g. [16], [32], [87], [88]). A detailed description of the Morlet wavelet approach applied here can be found elsewhere [5], [85]. In order to identify the latency and frequency range of the induced gamma amplitude peak, mean baseline-corrected spectral amplitudes (baseline: −200 to −50 ms prior to stimulus onset) across the two experimental conditions and the parieto-occipital electrodes used before (cf. Section A) were represented in a TF plot in the 30–90 Hz range. For further statistical analysis, the same time window as before (cf. Section A) covering maximal gamma amplitudes (150–400 ms after stimulus-onset) and the posterior regional mean were analyzed by using a paired t-test (familiar versus unfamiliar). Due to inter-individual differences in the gamma peak frequency, the wavelet designed for the frequency of the subject's maximal amplitude in the gamma range was chosen (mean peak frequency range in the 150–400 ms interval: 51 Hz; std error±12.9 Hz).

### Data analysis (C): reconstruction of the generators of the induced GBRs in source space

Intracranial current density distributions compatible with the observed scalp voltage topographies were estimated by means of variable-resolution-electromagnetic-tomography, VARETA [21], [22]. The software for source reconstruction was developed by some of the authors. This approach is explained in detail in Gruber et al. (2006) [20]. In brief, single trial VARETA analyses for a given frequency and time window were calculated in order to estimate the primary current densities that generate the measured iGBR peak. The conductor model was based on 3244 grid points (7.00 mm grid spacing), which were placed in registration with the recording area (128 electrodes) based on the average probabilistic MRI atlas (‘average brain’) produced by the Montreal Neurological Institute [89].

In order to localize differences in activation between the two conditions, statistical comparisons were carried out by means of a dependent ANOVA one-way statistical design (familiar versus unfamiliar) for the time window as defined in (A) and (B), i.e. 150–00 ms after stimulus onset. The outcome of the one-way ANOVA was used to construct corresponding statistical parametric maps (SPMs). To account for spatial dependencies between voxels activation threshold correction was calculated by means of Random Field Theory [90]. All results were depicted as 3D activation images constructed on the basis of the average Montreal brain [89]. Finally, regions of interests (ROIs) were defined by selecting voxels corresponding to cortical areas that showed significant differences in the gamma-band range. For subsequent coupling analysis, the voxel with maximal effect within each ROI was used (see Section D and E).

### Data analysis (D): long-range synchronization (phase-locking analysis) in source space

For each ROI (see Section C) and each trial the inverse solution was calculated in the time domain. In the following, the results obtained at each ROI were decomposed by PCA into their principal components, from which the first principal components were used for coupling analysis. Subsequently, phase synchrony analysis was performed, elaborating on a procedure suggested by several authors [14], [18], [91]. A detailed description of the whole procedure can be found elsewhere (e.g. [20], [40]). In brief, for each subject, phase synchrony was computed for the PCA-derived signal in a distinct frequency *f_0_* of his/her maximal gamma activity (f0±3 Hz; see also [18]) extracting the phase values by means of Morlet wavelet analysis. To examine whether a specific phase-locking value in the time window of maximal gamma power increase is statistical significant, a randomization technique was used. Within this time window, phase-locking values between all paired ROIs were calculated and averaged across subjects. In addition, 2000 PLV averages were analogously computed on shuffled data. Shuffling was performed by randomizing the order of trials and, then, calculating phase-locking between events not recorded at the same time. The average PLV was considered as statistically significant if it was greater than the maximum of the 2000 shuffled values, therefore indicating a probability value of p<0.001. To illustrate the results any significant phase-locking was depicted by a line from ROI *i* to ROI *j*.

To conclude with a methodological aspect of source reconstruction, it should be noted that our results regarding PLA in source space closely resembles the results of a previous publication [20]. However, in the study by Gruber and co-workers, PLA was calculated for separate forward solutions based on each iGBRs source in order to overcome the problem that each current source density consists of three directions (X, Y and Z). This would result in 3×3 possible couplings in source space. Here, we calculated our coupling measures directly between sources by using the first principal component of the signal at each of the three directions. Thus, this approach is capable to overcome the ‘three directions problem’ when using PLA and PDC in source space.

### Data analysis (E): information transfer (partial-directed coherence) in source space

In order to analyze information transfer between the identified regions of interest in source space, we computed partial-directed-coherence (PDC) on the same signals as used in section D. This coupling measure is based on multivariate-autoregressive (MVAR) modeling that simultaneously models spatial and temporal correlations, thus providing a spatio-temporal model of multi-sited brain signals [26], [57], [74]. In mathematical terms, the frequency-specific connectivity revealed by PDC is a realization of the concept of Granger-causality, according to which an observed time series *x*(*t_n_*) “Granger-causes” another series y(*t_n_*) at time instant *t_n_*, if knowledge of the past values of *x*(*t_n_*) significantly improves prediction of y(*t_n_*) [27], [92]. This relationship between time series is not reciprocal, i.e. *x*(*t_n_*) may *cause* y(*t_n_*) without y(*t_n_*) necessarily *causing x*(*t_n_*). This lack of reciprocity (or symmetry) allows to assess the direction of information transfer and, thus, to evaluate bidirectional coupling or feedback relationship.

Specifically, a multivariate autoregressive (MVAR) model was fitted to the time series revealed by the inverse solution at each ROI. To that end, the autoregressive model that was defined above for the univariate case (see Equation 1) has to be extended for the multivariate case (with 1 to M number of time series/ROIs) according to:
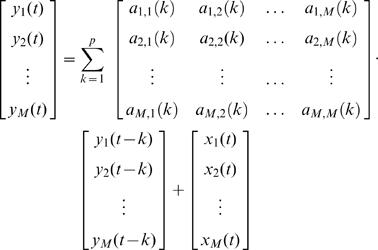
(3)This equation can be rewritten in matrix form as
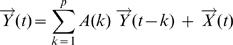
(4)The vector *Y*(*t*) represents the measured values (samples) for each of the *M* time series (M number of ROIs) at time instance *t*. The autoregressive parameters of all ROI combinations at time lag *k* form the matrices *A*(*k*) up to an order *p,* i.e. *A(1), A(2), … , A(p)* each with its *M*-by-*M* dimensionality. The off-diagonal elements of the multivariate AR parameter-matrix are the weighting factors defining the cross-terms between the ROIs.

Exemplary, the weighting factor *a*_1,*M*_(*k*) characterizes the contribution of ROI *M* to ROI *1* at time lag *k*. Finally, the vector *X*(*t*) represents the innovation process (cp. Section A) assumed to be a multivariate zero-mean white noise process. To uncover the spectral properties of the multivariate time series this model equation (Eq. 4) is transformed to the frequency domain yielding
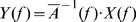
(5), where
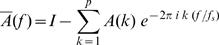
(6), with matrix *A̅*(*f*) representing the frequency-transformed AR-coefficients *a̅*_1,1_(*f*), *a̅*_1,2_(*f*), … , *a̅*_1,*M*_(*f*) up to *a̅* *_M_*_,*M*_(*f*), and with *f_S_* being the sampling frequency. The parameter *I* refers to the identity-matrix with a dimensionality of M by M.

On the basis of the frequency transformed multivariate parameters subsumed under *A̅*(*f*) the directed information transfer from ROI *j* to ROI *i* is quantified by partial-directed-coherence (PDC) as follows:
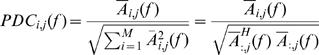
(7)with *A̅* *_i_*_,*j*_(*f*) being the *i*, *j-*th element and *A̅*_:,*j*_(*f*) the *j*-th column of the AR matrix *A̅*(*f*). The superscript *H* indicates the *Hermetian* operator, i.e. the transposed complex conjugate of matrix *A̅*(*f*). With this definition the frequency-specific causal influence of the time series from ROI *j* to ROI *i* is quantified in relation to all other information flow originating from ROI *j*. In other words, the PDC values obtained are normalized in respect to all the outflows from the source ROI *j* and range from zero to one, with one being the maximal level of information flow transmitted (0≥|*PDC_i,j_(f)*|^2^≤1). Additionally, the summed strength of all connections originating from ROI j, such as *PDC_1,j_(f)*, *PDC_2,j_(f)*, … *PDC_M,j_(f)*, is equal to one: 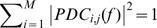 [26], [57].

Identical to the PLA approach (see Section D), the obtained signals at each ROI entered this analysis in the time window that covers maximal gamma amplitudes (150–400 ms after stimulus onset). The data were windowed in 128 samples-long intervals (i.e. 250 ms in length) and concatenated in one data stream per condition (as described in Section A). Finally, the PDC values were evaluated in the frequency band of the induced gamma peak (i.e. 40–90 Hz, see Results).

Since the distribution of PDC estimators is analytically not well established [27], [93], [94], we used the Jackknife approach for further statistical analyses [95], [96]. For details of this method we refer to [74], [96]. The threshold for our jackknife procedure was set to p<0.001. On an anatomical template of the ROI locations, each arrow pointing from the source ROI *i* to its target ROI *j* represents significant PDC.
